# Metal-Based Radiopharmaceuticals in Inorganic Chemistry

**DOI:** 10.3390/molecules28052290

**Published:** 2023-03-01

**Authors:** Alessandra Boschi, Petra Martini

**Affiliations:** 1 Department of Chemical, Pharmaceutical and Agricultural Sciences, University of Ferrara, 44121 Ferrara, Italy; 2 Department of Environmental and Prevention Sciences, University of Ferrara, 44121 Ferrara, Italy

The field of radiopharmaceuticals is constantly evolving thanks to the great contribution of specialists coming from different disciplines such as inorganic chemistry, radiochemistry, organic and biochemistry, pharmacology, nuclear medicine, physics, etc. In particular, the use of radiometals has experienced a great increase as a result of the development of radionuclides production technologies. In this particular area, inorganic chemistry skills are mainly involved in developing target-specific radiopharmaceuticals based on radiometals for non-invasive disease detection and cancer radiotherapy.

The Special Issue “Metal-Based Radiopharmaceuticals in Inorganic Chemistry”, which follows a similar topical Special Issue “New Trends in Production and Applications of Metal Radionuclides for Nuclear Medicine” [1,2,3,4,5,6,7,8,9,10,11,12,13,14,15,16], includes eleven research articles and one review. The production and applications of conventional and newly emerging research radiometals for diagnosis, therapy, and theranostics are the main focus of this Special Issue. Their employment in all Nuclear Medicine branches (SPECT/PET diagnostic, therapy, and theranostics) is regulated by their physical characteristics, such as half-life, radiation emission energy and type (γ, β^+^, β^−^, auger, α), availability and chemical ability to coordinate with ligands. The actual trend in the nuclear medicine research field is the use of radiometals for PET and SPECT, such as ^68^Ga, ^64^Cu, ^89^Zr, ^44^Sc, ^86^Y, ^52^Mn, ^99^mTc, etc., for therapy, such as ^177^Lu, ^90^Y, ^89^Sr, ^223^Ra, ^225^Ac, etc., and for theranostics, such as ^67^Cu, ^47^Sc, theranostics pairs, etc.

Hernández-Jiménez et al. [17] developed a ^225^Ac-delivering nanosystem by encapsulating the radionuclide into rHDL nanoparticles, as a potential targeted radiotherapeutic agent. It is well known that reconstituted high-density lipoproteins (rHDL) specifically recognize the scavenger receptor B type I (SR-BI) overexpressed in several types of cancer cells. Furthermore, after rHDL-SR-BI recognition, the rHDL content is injected into the cell cytoplasm. They performed the synthesis of rHDL in two steps using the microfluidic synthesis method for the subsequent encapsulation of ^225^Ac, previously complexed to the lipophilic molecule ^225^Ac-DOTA-benzene-p-SCN. The nanosystem (13 nm particle size) showed a radiochemical purity higher than 99% and stability in human serum. In vitro studies in HEP-G2 and PC-3 cancer cells (SR-BI positive) demonstrated that ^225^Ac was successfully internalized into the cytoplasm of cells, delivering high radiation doses to cell nuclei (107 Gy to PC-3 and 161 Gy to HEP-G2 nuclei at 24 h), resulting in a significant decrease in cell viability down to 3.22 ± 0.72% for the PC-3 and to 1.79 ± 0.23% for HEP-G2 at 192 h after 225Ac-rHDL treatment. After intratumoral ^225^Ac-rHDL administration in mice bearing HEP-G2 tumors, the biokinetic profile showed significant retention of radioactivity in the tumor masses (90.16 ± 2.52% of the injected activity), which generated ablative radiation doses (649 Gy/MBq). The results demonstrated adequate properties of rHDL as a stable carrier for selective deposition of ^225^Ac within cancer cells overexpressing SR-BI.

The work by Da Silva et al. [18] has been dedicated to developing new biomedical cyclotron irradiation and radiochemical isolation methods to produce ^165^Er suitable for targeted radionuclide therapeutic studies and characterize a new agent targeting prostate-specific membrane antigen. They irradiated 80–180 mg ^nat^Ho targets with 40 μA of 11–12.5 MeV protons to produce ^165^Er at 20–30 MBq·μA^−1^·h^−1^. Radiochemical isolation yielded ^165^Er in 0.01 M HCl (400 μL) with a decay-corrected (DC) yield of 64 ± 2%. Proof-of-concept radiolabeling studies were successfully performed synthesizing [^165^Er]PSMA-617, which will be utilized in vitro and in vivo to understand the role of AEs in PSMA-targeted radionuclide therapy of prostate cancer.

Helal et al. [19] proposed the use of ^213^Bi, an alpha-emitter with a short physical half-life of 46 min, to treat invasive fungal infections (IFI) with radioimmunotherapy (RIT). RIT uses antigen–antibody interaction to deliver sufficient activities of ionizing radiation to cells to induce DNA double-strand breaks and alter cell membrane and intracellular components for cell apoptosis while preserving healthy tissues. Microorganism-specific monoclonal antibodies have shown promising results in the experimental treatment of fungal, bacterial, and viral infections, including their recent and encouraging results from treating mice infected with *Blastomyces dermatitidis* with ^213^Bi-labeled antibody 400-2 to (1→3)-β-glucan. They performed a safety study of ^213^Bi-400-2 antibody in healthy dogs as a prelude for a clinical trial in companion dogs with acquired invasive fungal infections and later on in human patients with IFI. No significant acute or long-term side effects were observed after radioimmunoterapy (RIT) injections; only a few parameters were mildly and transiently outside reference change value limits, and a transient atypical morphology was observed in the circulating lymphocyte population of two dogs. Their results demonstrate the safety of systemic ^213^Bi-400-2 administration in dogs and encourage to pursuit of evaluation of RIT of IFI in companion dogs.

Summer et al. [20] developed one imaging probe for hybrid imaging combining the beneficial properties of radioactivity and optical imaging. They modified the macrocyclic gallium-68 chelator fusarinine C (FSC) by conjugating a fluorescent moiety and tetrazine (Tz) moieties. The resulting hybrid imaging agents were used for pretargeting applications utilizing click reactions with a *trans*-cyclooctene (TCO) tagged targeting vector for a proof of principle both in vitro and in vivo. The evaluation included fluorescence microscopy, binding studies, logD, protein binding, in vivo biodistribution, μPET (micro-positron emission tomography), and optical imaging (OI) studies. ^68^Ga-labeled conjugates showed suitable hydrophilicity, high stability, and specific targeting properties towards Rituximab-TCO pre-treated CD20 expressing Raji cells. Biodistribution studies showed fast clearance and low accumulation in non-targeted organs for both SulfoCy5- and IRDye800CW-conjugates. In an alendronate-TCO based bone targeting model the dimeric IRDye800CW-conjugate resulted in specific targeting using PET and OI, superior to the monomer. This proof-of-concept study showed that the preparation of FSC-Tz hybrid imaging agents for pretargeting applications is feasible, making such compounds suitable for hybrid imaging applications.

Two research articles covered the production and preparation of PET radionuclides and radiopharmaceuticals. Kazakov et al. [21] have dedicated a study to cobalt ^55^Co, ^57^Co, and ^58m^Co isotopes production. These are considered to be promising radionuclides in nuclear medicine, with ^55^Co receiving the most attention as an isotope for diagnostics by positron emission tomography. They determined the yields of nuclear reactions occurring during the irradiation of ^nat^Ni and ^60^Ni by bremsstrahlung photons with energy up to 55 MeV and developed a method of fast and simple cobalt isotopes separation from irradiated targets using extraction chromatography. Targets made of ^nat^Ni and ^60^Ni were irradiated by bremsstrahlung photons with energy up to 55 MeV. They found that in every case, the activities produced of ^56,57,58^Co were higher than the activity of ^55^Co, and therefore enough for preclinical research of radiopharmaceuticals based on cobalt. They also demonstrated that the radionuclide purity of ^55^Co produced by the photonuclear method at 55 MeV is not sufficient for PET. They demonstrated that the separation of Co(II) is possible in a wide range of HCl concentrations (from 0.01 to 3 M). The separation factor of Ni/Co was 2.8·× 10^5^, the yield of Co(II) was close to quantitative, and separation lasted for no longer than 0.5 h.

Another radionuclide of great interest is the PET radionuclide zirconium-89 (^89^Zr) and the increased interest in immunoPET imaging probes for preclinical and clinical studies has led to a rising demand for this radionuclide. ^89^Zr emits highly penetrating 511 and 909 keV photons delivering an undesirably high radiation dose, which makes it difficult to produce large amounts manually. Considering also the growing demand for Good Manufacturing Practices (GMP)-grade radionuclides for clinical applications Gaja et al. [22] in their study have adopted the commercially available TRASIS mini AllinOne automated synthesis unit to achieve efficient and reproducible batches of ^89^Zr. The automated module is used for the target dissolution and separation of ^89^Zr from the yttrium target material. ^89^Zr is eluted with a very small volume of oxalic acid (1.5 mL) directly over the sterile filter into the final vial. Using this sophisticated automated purification method, they obtained a satisfactory amount of ^89^Zr in high radionuclidic and radiochemical purities of over 99.99%. The specific activity of three production batches was calculated and was found to be in the range of 1351–2323 MBq/μmol. ICP-MS analysis of final solutions showed impurity levels always below 1 ppm.

Recently, the technological advancement in the radionuclides cyclotron-based production sector has encouraged the use of novel radioisotopes (mainly radiometals) in medical applications, for implementing the so-called personalized medicine approach. The availability of cyclotron-produced radiometals requires the use of solid targets and a solid target dissolution system and in this context fits the study of Sciacca et al. [23]. They developed a simple and efficient solid target dissolution system compatible with commercial cassette-based synthesis modules. In this way, it would be possible to perform the radiochemical processing, from the dissolution to the labeling, all at once using a single remotely controlled device. Keeping the system compact allows for containing all the processes in a single hot cell, lowering the probability of external and operator contamination. At the same time, this reduces the processing time and maximizes the recovery yield thanks to the absence of wasteful transfers from one system to another. The entire process, starting from dissolution up to radiopharmaceutical formulation, can be applied continuously.

The presented solid target dissolution system concept relies on an open-bottomed vial positioned upon a target coin. In particular, the idea is to use the movement mechanism of a syringe pump to position the vial up and down on the target and to exploit the heater/cooler reactor of the module as a target holder. All the steps can be remotely controlled and are incorporated in the cassette manifold together with the purification and radiolabelling steps.

In their study, Štícha et al. [24] have successfully adapted the Schotten–Baumann (SB) reaction for the derivatization of MS hardly ionizable Re(VII) chlorocomplexes.

Mass spectrometry helps to ensure the chemical identity and desired purity of the prepared complexes before their medical application, and plays an indispensable role in clinical practice. In the context of mass spectrometry, specific problems arise with the low ionization efficiency of particular analytes. Chemical derivatization was used as one of the most effective methods to improve the analyte’s response and separation characteristics. They studied the reaction of the Re(VII) bis(catechol) chlorocomplex with the set of halogen and alkyl anilines as derivatization agents and found that the SB reaction products are easily ionizable under common ESI conditions providing structurally characteristic molecular and fragment anions. Based on DFT computation, the effect of Re-N bond shortening in the course of complex deprotonation was simulated and also correlated with the basicity of the aniline derivative used as a derivatization agent. Their conclusions follow the known relation between the basicity of the reaction environment and the yield of the SB reaction. However, an attempt to increase the yield of the derivatization reaction by adding triethylamine (TEA) to the reaction mixture was unsuccessful. Such a conclusion probably refers to the fast reaction providing the dioxorhenium complex as a competition to the SB reaction itself.

A research article reported the development of a solvent extraction and separation process of technetium from molybdenum in a micro-scale in-flow chemistry regime with the aid of a capillary loop and a membrane-based separator, respectively [25]. The developed system can extract and separate quantitatively and selectively (91.0 ± 1.8% decay corrected) the [^99m^Tc]TcO_4_Na in about 20 min, by using a ZAIPUT separator device. In this work the authors demonstrated for the first time, the high efficiency of a MEK-based solvent extraction process of ^99m^Tc from a molybdenum-based liquid phased in an in-flow micro-scale regime. This system allows the extraction time and separation of technetium from the organic phase to be drastically reduced and can be used both to purify technetium from molybdenum metal targets in the direct cyclotron ^99m^Tc production, as well as in indirect ^99m^Tc production such as the ^99^Mo production, by irradiating natural molybdenum using a 14-MeV accelerator-driven neutron source.

Remaining within the scope of SPECT, a specific work has been dedicated to indium-111 [26]. In this study, a CCK-2R-targeting ligand based on the nastorazepide core was syn- thetized and functionalized with a DOTA chelator with the aim to provide a suitable platform for a theragnostic approach with radioactive metals. Avoiding the use of peptide- based sequences in the structure, including the linker, allowed them to obtain a molecule with high stability in physiological media that could be easily labeled with indium-111 as a pivotal radionuclide for future studies. The obtained radiotracer was successfully employed in the imaging of CCK-2R-expressing xenograft tumors in mice, but further structural studies are needed for enhancing receptor affinity and biodistribution. Additionally, the presented results are particularly noteworthy since the ability of a targeting probe to image cancers has been demonstrated using human cells expressing physiological levels of CCK-2R instead of transfected cells like the majority of the studies on the topic so far.

The objective of the research presented by Blumberg et al. [27] was to evaluate and compare different open-chain and bridged p-tert-butylcalix [4] arene derivatives as possible leading compounds that could, upon further modification, yield viable chelators for the selected divalent metal ions Sr^2+^, Ba^2+^, and Pb^2+^ in radiopharmaceutical applications and provide information about comparable stability constants. UV titration as a reliable and constant method for the calculation of stability constants was used to determine association constants for the respective ions. Additionally, theoretical calculations involving Ba^2+^ as a surrogate for Ra^2+^ were accomplished to underline the results. They found that the additional proton-ionizable side functions connected with a benzocrown ether structure to the calixarene skeleton led to a considerable improvement of the complex stability resulting in high association constants.

Finally, the only review of the Special Issue covered ^67^Cu production capabilities [28]. This review reveals the international effort to supply ^67^Cu, a promising theranostic radionuclide. The increasing availability of intense particle accelerators and the optimization of the associated technologies, targetry, and radiochemical processing, are making ^67^Cu closer to the clinics. In particular, ^64^Cu is now widely available for clinical use, promoting the development of innovative Cu-labeled radiopharmaceuticals. The improved availability of ^67^Cu would speed up further radiopharmaceutical applications for therapy. In addition to a detailed analysis of the possible nuclear reactions to produce ^67^Cu, the radiochemical procedures to extract and purify Cu from the bulk material were also described in this work. Recent developments in the photoproduction of ^67^Cu, and in the possibility of having accelerators providing intense 70 MeV proton beams and/or intense 30 MeV deuteron beams, are grounds for a future reliable supply of ^67^Cu.
